# Seasonal Changes in Qualitative and Quantitative Characteristics of Humic Substances in Waters of Different Genesis: Membrane Technologies and Equilibrium Processes

**DOI:** 10.3390/membranes13030340

**Published:** 2023-03-15

**Authors:** Marina Dinu

**Affiliations:** Vernadsky Institute of Geochemistry and Analytical Chemistry (GEOKHI RAS), 119334 Moscow, Russia; marinadinu999@gmail.com

**Keywords:** organic substances, zeta potential, molecular weight distribution, fractionation, metal ions, speciation

## Abstract

Membrane filtration methods were applied in this study to research natural waters specification (and speciation). Lysimetric waters (soil waters) of background territories in different seasons are considered. Features of the change in molecular weights, elemental composition, and zeta potential of organic matter during fractionation from 8 μm to 100 kDa were found. The number of labile and non-labile speciation of some elements obtained by membrane filtration and ion-exchange separation methods were found and compared. The highest molecular weights of organic substances were found in summer samples of lysimetric waters (more than 100 kDa) with a predominance of the aromatic component in the IR spectra of the samples. Several maxima were also found in the molecular weight distribution, including the increase in autochthonous organic substances. The most stable negative zeta potential, as a stabilized colloid matter, are represented in summer (near −26 mV) and in autumn (near −22 mV) lysimetric water. A slight increase in metal ions bound into organic complexes is typical for summer and autumn samples.

## 1. Introduction

The active technological development of membrane filtration, combined with the ease of execution and optimization of installation, has led to the fact that these methods are representative and common in the analysis of natural water. The greatest emphasis in the application of such methods is placed on the study of the element’s speciation in natural and industrial waters, as well as on methods for purifying process and municipal water from several pollutants. The literature also reflects some aspects of changing the quantity of organic substances using membrane filters and ion exchange resins as methods for their removal. Ion-exchange water purification is no less popular in terms of ease of implementation and quality of the result.

Nevertheless, there are practically no scientific works related to the use of membrane filtration for fundamental scientific research of environmental objects, for example, to analyze the quality and quantity of organic substances in natural waters [1,2].

At the same time, waters with high contents of organic substances are widespread in Russia, and the natural and climatic factors of the regions determine a sufficient variety of structural features and properties of these components [1,2]. This fact makes it quite relevant to study changes in some parameters of water organic substances by separation methods (membrane, resin).

Humic substances are characterized by high-molecular weight and mix structure because of complex biochemical processes. These mechanisms depend on specific landscape characteristics, climatic factors, and anthropogenic impact [1,3,4,5,6,7]. The contribution of biochemical processes to the formation of humic compounds is a controversial issue. A large number of scientists point to the dominant biological role of transformations at almost all stages of formation, and only the final stage is about the contribution of chemical processes [1,3,4,5,6,7]. In addition, natural macromolecular compounds are acids of various activities and are characterized by significant complexing potential in context with element ions in natural waters, which determines their protective role in the biosphere [2,7,8]. The complexes of metals with humus ligands formed in natural waters (humate and fulvate), as well as those complex compounds that came from the catchment area of the reservoir in the process of leaching, determine the biosphere level and its buffering property. There are a large number of works devoted to the study of the physicochemical characteristics of humic substances and the mechanisms of binding with metal ions, but there is no single research methodology at the moment [7,9,10,11].

Organic substances in waters are represented by compounds of various natures. It is considered possible to separate them into allochthonous and autochthonous components [12], for which ion exchange technique and electrolytic separation are used as the first stage of the preparation of samples from natural waters. Currently, directions are being developed for the synthesis and application of various types of polymer resins for the most complete and detailed separation of organic substances by molecular weights [13,14].

The autochthonous component is easily oxidized, with low molecular weight compounds (proteins, lipids), which are formed in the reservoir due to numerous biochemical processes. According to the literature data [15], their contribution to the chemical equilibrium is small and largely depends on the physicochemical conditions and biological processes. Allochthonous substances include humic compounds washed out from the watershed, as well as wastewater organic matter. As mentioned, their protective role is most significant for existing chemical equilibria due to pronounced acid–base and redox properties.

Questions of the structure and, of course, properties of humic organic substances have attracted scientists for more than a decade [4,6,7,16,17,18]. Particular attention is paid to soil humus (organic matter) contained in soils as a more concentrated sample for research. There is enough scientific material on the qualitative composition of humic substances (HS) in soils, but due to the problems of zonal and seasonal variability structures, the properties of natural waters' organic matter remain poorly understood.

**Figure 1:**
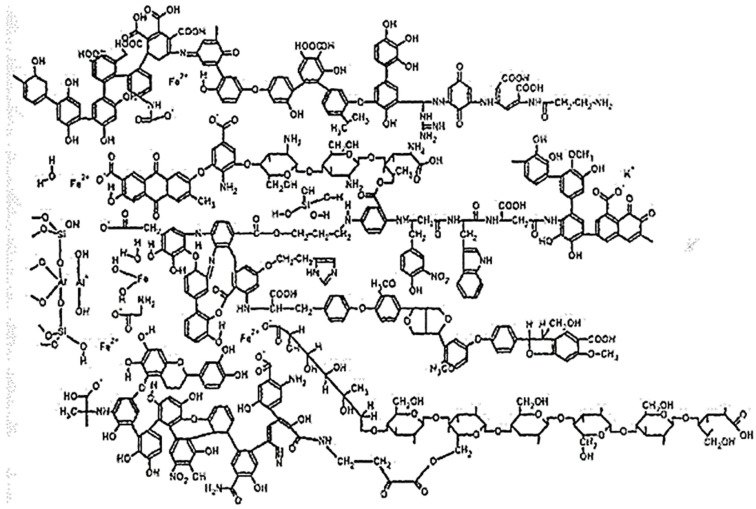
The most general visualization of the structure of organic humic substances [3,4].

It is known from recent works [19,20] that allochthonous humic substances in natural waters are supramolecular associates of heterogeneous molecules bound by hydrophobic interactions (van der Waals forces, π-π and ion-dipole bonds) and hydrogen bonds (Figure 1). All these forces stabilize the structure of molecular aggregates, although it is impossible to determine exactly to what extent each bond predominates. One can only assert that different levels and types of interactions form supramolecular structures of an organic conglomerate with individual physicochemical properties. However, there is no evidence-based structure of humic substances. The most generally accepted structure, reflecting the possible chemical properties of substances, is shown in Figure 1.

The stability of HS globules varies depending on the ionic strength of the solution and pH [1]. The supramolecular structures of humic substances, which are currently receiving sufficient attention, make it possible to verify the differences in the structure of organic substances under different conditions. For example, the work [11] shows of five possible levels of organization of humic substances depending on the biogeochemical nature. It should be noted that each level (or subfractions) of aqueous humic substances has a specific set of physicochemical characteristics (MM, zeta potential, and size), as well as a different ability to interact with toxicants (for example, metal ions and toxic organics).

Changes in parameters such as zeta potential, molecular weight, and colloid size in combination with IR spectroscopy and chromate-mass spectra can express indicators of the organic substance quality in the process of their separation. 

The research aim was a detailed study of the structural features of natural aqueous organic substances and their protective properties using modern express technology of membrane filtration and ion-exchange separation.

## 2. Materials and Methods

### 2.1. Objects and Methods

The studied points (lysimetric waters) are distant from local human-caused impacts (protected territory of the Russian Federation). The city of Valdai is located more than 100 km from large industrial centers and does not have a developed industry [8,9,16,21]. Despite this, local urban infrastructures and possible transboundary migrations of pollutants can influence on the chemical composition of water [5].

**Figure 2:**
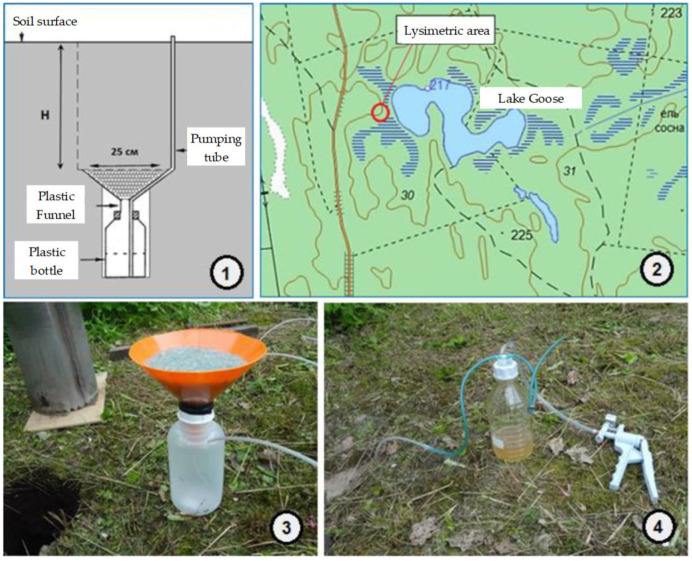
Sampling scheme: (**1**) scheme of the lysimeter; (**2**) location of the lysimetric platform; (**3**) lysimeter; (**4**) system for manual pumping of lysimetric waters.

Research was carried out in 2015–2022 (spring, summer, and autumn samples) (Figure 2). We installed lysimetric equipment in sub-humus depths to take into account the influence of genetic horizons on the composition of soil waters in 2015. In the studies, leaf-type lysimetric installations were used. The main advantage of choosing such installations is to create conditions as close to natural as possible. The use of a soil monolith as a lysimeter body does not disturb the hydrodynamic regime. Therefore, the real amount of gravitational moisture located at a certain depth of the profile is taken from the lysimeters. The disadvantage of the method is the impossibility of determining the volume of the soil substrate with which the soil solution interacted. The soil water filtrate was pumped out with a vacuum pump through an outlet tube. We measured in situ pH and temperature (T) (in situ) using a pH Mettler Tolledo instrument (Mettler Tolledo Switzerland, Greifensee, Switzerland).

In a short time, aqueous samples were transported in inert plastic bottles and analyzed after filtration through a 0.45 µm Millipore membrane filter. The elemental composition was determined by inductively coupled plasma atomic emission spectrometry (iCAP-6500, Thermo Scientific, Waltham, MA, USA) and inductively coupled plasma mass spectrometry (X-7, Thermo Scientific, Waltham, MA, USA). The correctness of the results was achieved by 10% (or less) by the discrepancy between the cationic–anionic composition.

The main analytical criteria were studied by the following methods: potentiometric and titrimetric, using spectrophotometry, and anionic–cationic composition (calcium, magnesium, potassium, sodium, ammonium ions, sulfates, chlorides, fluorides, nitrates, nitrites, and ortho-/polyphosphates) by ion chromatography methods, as well as voltammetric and atomic absorption methods.

### 2.2. Membrane Filtration and Ion Exchange Separation of Samples

Separation of water aliquots was performed near the sampling point. Syringe-ring filter nozzles and a multi-stage tangential membrane filter unit were used [9]. Membrane pore sizes were standard for this study: 8 µm, 1.2 µm, 0.45 µm, and 0.2 µm (VLADiSART). The initial sample volume was at least 250 mL. Next, we performed sequential filtration of the initial samples. A 5 mL aliquot was separated from each filtrate fraction for elemental analysis by inductively coupled plasma mass spectrometry. At the installation of multistage tangential membrane filtration, the sample was passed through the same set of membranes and analyzed solutions were taken. The mass balance of all metals was controlled at each stage of fractionation (difference no more than 15%). We evaluated the following possible material components of the solution: mechanical suspension and oxidized impurities (>8 µm, 1.2 µm, 0.45 µm, and 0.2 µm).

In the course of experimental work, the content of metals in suspended (unfiltered) and dissolved (filtered) fractions was determined. They were further separated into fixed and non-fixed using an ion-exchange resin—strongly bound to the organic matter of natural waters (Dowex 50 W-X8, 50–100 mesh in Na+ form). That is, conditionally labile and non-labile according to the chosen ion exchange [10,11]. The labile form of the presence of elements is aquaionic, bonding with inorganic (including mixed) metal complexes and weak complexes with organic matter (with a low conditional stability constant). The non-labile forms included strong metal complexes with organic matter, predominantly of a humic nature.

The change in the set of functional groups was carried out using IR spectral analysis and chromatography–mass spectra (Thermo instruments, Agilent, Santa Clara, CA, USA). The study of the parameters of organic substances was carried out after freeze drying and freezing on a Zeta-sizen-nano device (Melvern). The separation of organic substances into allochthonous and autochthonous components was carried out using cellulose, followed by determination of the functional characteristics of each sample.

### 2.3. Statistical Analysis

Statistic Advanced 13 software was used to analyze multivariate data. Discriminant analysis with canonical visualization was used to determine group differences. Correlation analysis was used to assess strong significant relationships.

## 3. Results and Discussion

### 3.1. Basic Hydrochemical Parameters

According to Table 1, some hydrochemical compositions of waters can be characterized. Lysimetric waters are characterized by a slightly acidic pH value, with minimum average values of 4.3 for the autumn season, which is associated with the end of the growing season. The content of organic matter is highest in the warmest summer period, when, in addition to allochthonous organic matter, allochthonous organic matter is added. Electrical conductivity varies (on average) from 50 µS/cm to 21 µS/cm and is highest in the spring after snow melt (melt water runoff). The content of a number of trace elements varies by 1.5–2 times, for example, for iron and aluminum ions. The greatest changes are typical for zinc and copper ions, which are characterized by geochemical factors of entry into lysimetric waters.

### 3.2. Seasonal Physical and Chemical Changes in the Properties of Organic Natural Substances

According to Table 2, Table 3 and Table 4, a change in a number of parameters of organic substances was revealed. We can say that the most stable state of the colloid is characteristic of the summer months. The value of the zeta potential varies from −23 to −25 mV, and the molecular weights during this period are max in the summer period and are more than 1000 kDa. These values are affected by greater leaching from the watershed and the formation of bonds with intra-aquatic organic substances. In the same summer period, the scatter of the peaks responsible for particle sizes is clearly visible—from three to four stable peaks characteristic of organic substances of both allochthonous and autochthonous types, i.e., the contribution of biological processes is the greatest.

**Figure 3:**
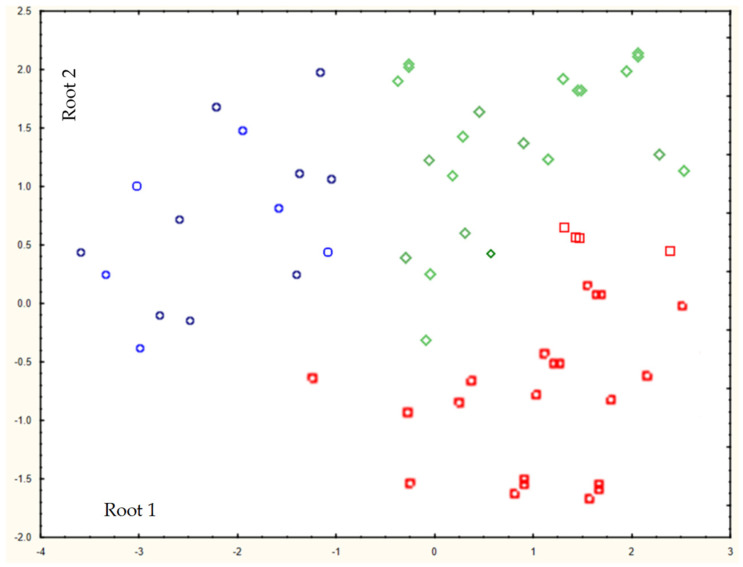
Discriminant analysis with canonical visualization. The blue, red, and green colors of the markers represent spring, summer, and autumn, respectively.

In the spring months (Table 2), the so-called dilution effect of the solution appears. The leaching of allochthonous organic substances from soils into lysimetric waters is slow in the winter season and the ingress of melted snow water contributes to a low content of organic substances—in mg O/l and total C and a rather unstable state of the colloid (sometimes above −20 mV). The distribution of sizes and molecular weights of organic substances is the smallest (two peaks rarely appear). During the summer period, the samples are characterized by the most negative zeta potentials and the maximum molecular weights of organic substances, as found (Figure 3).

Autumn (Table 4) samples of lysimetric waters are also characterized by a stable negative charge of the zeta potential, but not as low as for summer samples. The distribution of sizes and molecular weights in autumn samples is not so diverse (2–3 peaks) and is characterized by the predominance of a smaller range of sizes.

### 3.3. Application of Membrane Filtration and Ion Exchange Separation to Evaluate the Properties of Organic Substances

The first step that the two methods aim to accomplish is the separation of the whole sample into its component parts. If the 1st method is primarily associated with the identification of different-sized elements speciation, and only then makes it possible to investigate changes in the state of qualitative features of humic substances, then the 2nd one is completely associated with a change in the proportion of bound/free (ionic) ionic forms as a conditional characteristic of the formation of equilibrium in the system [19,20,21,22,23].

Nevertheless, both of these methods, in combination with other research methods, make it possible to assess both the material state of elements (metals in the first place) and changes in the physicochemical characteristics of organic substances in water. There are a number of works criticizing the use of membrane filters for assessing the state of the environment, since filters fix a number of organic components and reduce the reliability of the experiment. Nevertheless, a sufficient number of works indicate the systemic, express use of these methods.

The parameters characterizing the organic substances of natural waters when they are separated into specification (membrane separation and ion-exchange separation) are zeta potential, molecular weight, and compound size, making it possible to identify the most important seasonal factors. According to Figure 3, a statistically significant separation was revealed according to the following discriminating parameters: molecular weight (F = 10), zeta potential (F = 18), and ratio of aromatic/aliphatic groups (F = 8).

**Figure 4:**
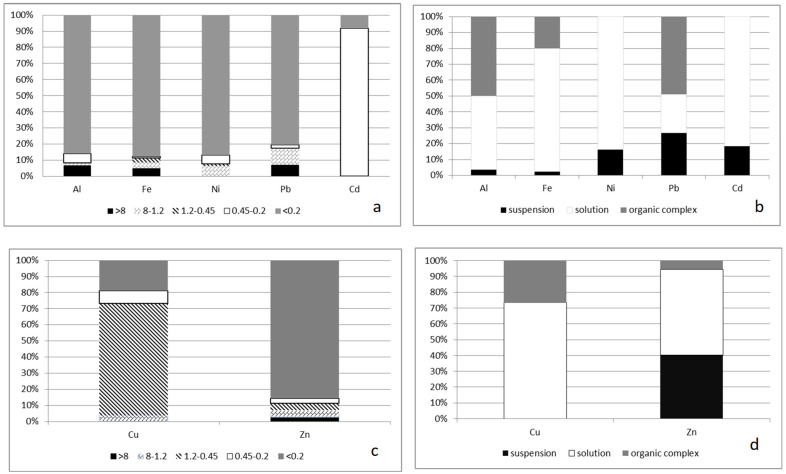
Dispersion composition and labile/non-labile specifications of elements in water samples in spring: (**a**,**b**) this is a membrane separation method; (**c**,**d**) this is an ion exchange separation method.

**Figure 5:**
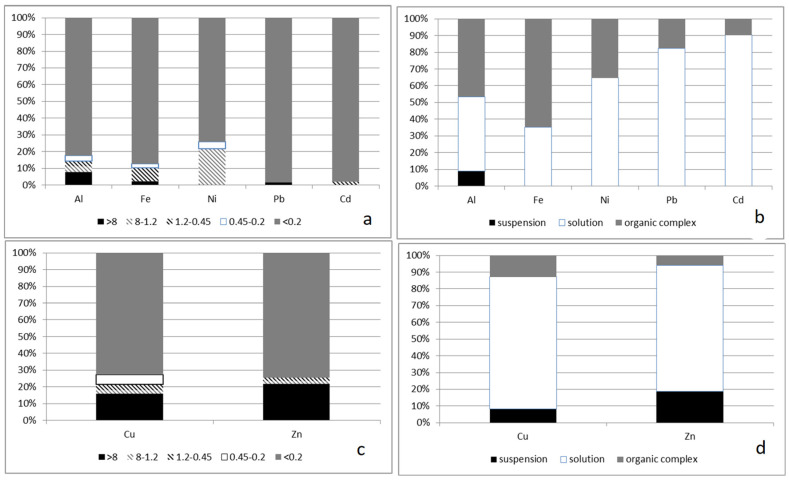
Dispersion composition and labile/non-labile specifications of elements in water samples in summer: (**a**,**b**) this is a membrane separation method; (**c**,**d**) this is an ion exchange separation method (suspension—more than 0.45 microns).

**Figure 6:**
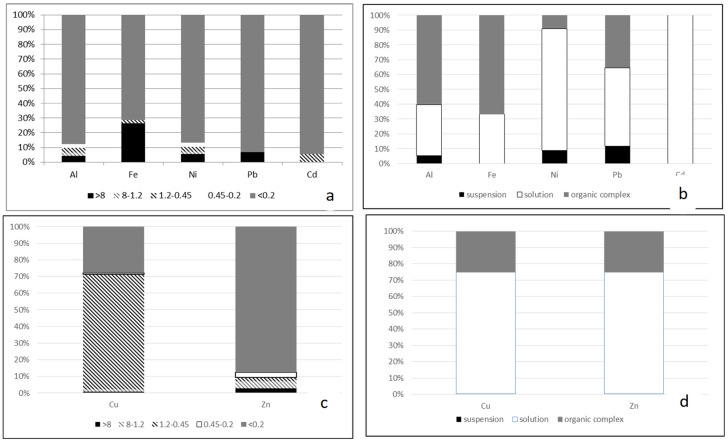
Dispersion composition and labile/non-labile specifications of elements in water samples in spring: (**a**,**b**) this is a membrane separation method; (**c**,**d**) this is an ion exchange separation method (suspension—more than 0.45 microns).

An increase in the stability of colloids (a decrease in the zeta potential) and an increase in the proportion of non-labile compounds of some metals (for example, iron), most pronounced in summer water samples (Figure 4, Figure 5 and Figure 6), was found.

The decrease in the size of colloids is also most significant for summer and autumn samples (Figure 4 and Figure 6), which is associated with the contribution of the biological component; at the same time, the balance of allochthonous/autochthonous organic substances is maximum in the summer–autumn period.

In general, in the studied range of temperature seasons, the change in the content of aromatic subfractions of organic substances is the largest and maximum in the summer period.

The molecular weight distribution in summer contains from three to six maxima, and the formation of complexes with metal ions is possible both through allochthonous organic substances and with the help of autochthonous organic substances. Comparison of two methods for studying organic substances (Figure 4) revealed a number of correspondences between their protective properties. For ions of iron, aluminum, and nickel (partially) in the summer–autumn period, soluble specification of the element (less than 0.45 microns) correlate with non-labile forms of the indicated elements. In the spring season, after the snow melts, for all ions of the selected elements, labile specification prevails as the correspondence to samples less than 0.45 microns.

The change in the quality of organic substances based on other spectral analyses indicates the formation of lower molecular weight complexes of metal ions (iron, aluminum, and copper) with organic substances in summer due to carbonyl and carboxyl groups; in autumn, after vegetation processes and biochemical transformations of humic substances, more coordinatively heavy metal complexes are characteristic [23,24,25,26,27].

## 4. Conclusions

Organic substances of humus nature play an important role in the formation of the chemical composition of waters and the occurrence of homogeneous and heterogeneous processes. The development of ideas about their protective properties is directly related to the increase in anthropogenic impact: the intake of heavy metal ions, organic synthetic toxicants, petroleum products, and radionuclides. Humic substances are able to bind and inactivate toxic elements and substances in natural objects. 

Experimental and published data point to the high promise of combining a number of methods of ion exchange technology and membrane filtration for the isolation and finer study of organic substances of a humic nature with different molecular weights and natures.

The data obtained by us indicate the possibility of identifying (corresponding) labile/non-labile forms of elements with the sizes of compounds of these elements.

The decrease in the size of colloids is also most significant for summer and autumn samples, which is associated with the contribution of the biological component; at the same time, the balance of allochthonous/autochthonous organic substances is at the maximum in the summer–autumn period. In general, in the studied range of temperature seasons, the change in the content and qualitative features of organic substances is the greatest and reaches the maximum in the summer period.

## Figures and Tables

**Table 1:** Physical and chemical parameters of water samples.

Parameter	Spring	Summer	Autumn
pH	6.0	5.5	4.3
	5.8–6.6	5.2–6.0	3.5–4.6
T, °C	12	17	6.0
	8.0–15	15–22	5.8–8.0
Conductivity, µS/cm	50	21	36
	22–105	18–24	29–42
Color, °	120	580	290
	100–300	300–670	220–340
permanganate oxidizability, mg O/L	160	300	20
	100–200	200–700	110–220
C org, mg/L	43	80	40
	39–47	72–100	35–50
Fe tot, mg/L	0.36	0.53	0.49
	0.15–0.90	0.32–0.99	0.41–0.55
Fe-Org, mg/L	0.29	0.19	0.42
	0.11–0.31	0.11–0.33	0.39–0.45
Cu tot, µg/L	516	1057	256
	420–568	820–1140	206–304
Cu-Org, µg/L	76	50	64
	59–88	42–84	59–76

**Table 2:** Physical and chemical parameters of organic substances in spring water samples.

Parameter	Spring Season									
	In the Original Solution	>8	8–1.2	1.2–0.45	0.45–0.2	<0.2	In the Original Solution	Suspension	Solution	Organic Complex
Size, nm	820.00	810.00	650.00	599.00	560.00	395.00	818.00	-	580.00	500.00
Zeta potential, mV	−10.00	−8.00	−11.00	−16.00	−19.00	−21.00	−12.00	−	−18.00	−25.00
Al, mg/L	1.07	0.07	0.02	0.00	0.06	0.92	1.05	0.04	0.49	0.53
Fe, mg/L	0.39	0.02	0.01	0.01	0.00	0.34	0.39	0.01	0.30	0.08
Cu, µg/L	187.00	0.00	7.00	130.00	15.00	35.00	187.20	0.20	137.00	50.00
Ni, µg/L	0.82	0.00	0.05	0.01	0.05	0.71	1.05	0.17	0.88	0.00
Pb, µg/L	0.05	0.00	0.01	0.00	0.00	0.04	0.49	0.13	0.12	0.24
Cd, µg/L	0.14	0.00	0.00	0.00	0.11	0.01	0.17	0.03	0.14	0.00
Zn, µg/L	257.54	7.09	10.99	11.13	7.91	220.41	254.21	102.40	137.73	14.08

**Table 3:** Physical and chemical parameters of organic substances in summer water samples.

Parameter	Summer Season									
	In the Original Solution	>8	8–1.2	1.2–0.45	0.45–0.2	<0.2	In the Original Solution	Suspension	Solution	Organic Complex
Size, nm	800.00	980.00	965.00	940.00	860.00	700.00	970.00	-	875.00	789.00
Zeta potential, mV	−24.00	−20.00	−23.00	−26.00	−28.00	−35.00	−18.00	−	−30.00	−30.00
Al, mg/L	1.55	0.12	0.05	0.05	0.06	1.28	1.55	0.14	0.69	0.72
Fe, mg/L	0.27	0.01	0.00	0.02	0.01	0.24	0.28	0.00	0.10	0.18
Cu, µg/L	367.7	58.90	7.26	12.86	21.59	267.05	367.00	29.90	290.60	47.10
Ni, µg/L	3.20	0.00	0.68	0.00	0.14	2.37	2.03	0.00	1.31	0.72
Pb, µg/L	3.12	0.04	0.00	0.00	0.00	3.08	1.52	0.00	1.26	0.27
Cd, µg/L	0.22	0.00	0.00	0.00	0.00	0.22	0.22	0.00	0.19	0.02
Zn, µg/L	1039.65	224.93	20.83	18.27	0.00	775.62	977.23	183.29	736.56	57.38

**Table 4:** Physical and chemical parameters of organic substances in autumn water samples.

Parameter	Autumn									
	In the Original Solution	>8	8–1.2	1.2–0.45	0.45–0.2	<0.2	In the Original Solution	Suspension	Solution	Organic Complex
Size, nm	680.00	687.00	620.00	598.00	402.00	340.00	690.00	-	396.00	345.00
Zeta potential, mV	−15.00	−12.00	−16.00	−18.00	−20.00	−24.00	−14.00	−	−15.00	−26.00
Al, mg/L	1.71	0.07	0.04	0.05	0.05	1.50	1.71	0.09	0.59	1.03
Fe, mg/L	0.18	0.05	0.00	0.00	0.00	0.13	0.10	0.00	0.03	0.06
Cu, µg/L	256.00	7.21	10.94	556.00	8.19	223.75	256.00	1.02	190.20	64.42
Ni, µg/L	0.70	0.04	0.01	0.02	0.02	0.61	0.70	0.06	0.58	0.06
Pb, µg/L	2.77	0.19	0.00	0.00	0.00	2.58	1.62	0.19	0.86	0.57
Cd, µg/L	1.48	0.00	0.00	0.08	0.00	1.39	0.10	0.00	0.10	0.00
Zn, µg/L	255.63	7.20	10.94	5.56	8.19	223.75	255.63	1.02	190.20	64.42

## Data Availability

Not applicable.
